# Bacterial Communities Associated with *Poa annua* Roots in Central European (Poland) and Antarctic Settings (King George Island)

**DOI:** 10.3390/microorganisms9040811

**Published:** 2021-04-12

**Authors:** Anna Znój, Jakub Grzesiak, Jan Gawor, Robert Gromadka, Katarzyna J. Chwedorzewska

**Affiliations:** 1 Department of Antarctic Biology, Institute of Biochemistry and Biophysics, Polish Academy of Sciences, Pawińskiego 5A, 02-106 Warsaw, Poland; aznoj@ibb.waw.pl; 2 Botanical Garden-Center for Biological Diversity Conservation, Polish Academy of Sciences, Prawdziwka 2, 02-973 Warsaw, Poland; 3 Environmental Laboratory of DNA Sequencing and Synthesis, Institute of Biochemistry and Biophysics, Polish Academy of Sciences, Pawińskiego 5A, 02-106 Warsaw, Poland; gaworj@wp.pl (J.G.); robert@ibb.waw.pl (R.G.); 4 Department of Agronomy, Warsaw University of Life Sciences-SGGW, Nowoursynowska 166, 02-787 Warsaw, Poland; kchwedorzewska@o2.pl

**Keywords:** rhizosphere, microbiome, endosphere, roots, invasive species

## Abstract

*Poa annua* (annual bluegrass) is one of the most ubiquitous grass species in the world. In isolated regions of maritime Antarctica, it has become an invasive organism threatening native tundra communities. In this study, we have explored and compared the rhizosphere and root-endosphere dwelling microbial community of *P. annua* specimens of maritime Antarctic and Central European origin in terms of bacterial phylogenetic diversity and microbial metabolic activity with a geochemical soil background. Our results show that the rhizospheric bacterial community was unique for each sampling site, yet the endosphere communities were similar to each other. However, key plant-associated bacterial taxa such as the *Rhizobiaceae* family were poorly represented in Antarctic samples, probably due to high salinity and heavy metal concentrations in the soil. Metabolic activity in the Antarctic material was considerably lower than in Central European samples. Antarctic root endosphere showed unusually high numbers of certain opportunistic bacterial groups, which proliferated due to low competition conditions. Thirteen bacterial families were recognized in this study to form a core microbiome of the *P. annua* root endosphere. The most numerous were the *Flavobacteriaceae*, suspected to be major contributors to the ecological success of annual bluegrass, especially in harsh, Antarctic conditions.

## 1. Introduction

*Poa annua* L. (annual bluegrass) is one of the most ubiquitous grass species in the world. It can be found growing on every continent, having established populations even in the high Arctic and Antarctica [1,2,3]. Its extensive adaptability to a wide range of environmental conditions makes it a stubborn weed and pioneer species. *P*. *annua* has developed a series of features that ensure its ecological success. It can deal with biotic and abiotic stressors as well as habitat instability by compact growth habit, a high fraction of biomass allocated into belowground organs, and the presence of both annual and perennial life forms [4,5,6]. It also displays huge phenotypic and genotypic variability, making it difficult in the past to determine its evolutionary origin [1].

These adaptive traits caused *P*. *annua* to be a highly competitive, invasive species in many isolated environments of unique biodiversity [7]. Maritime Antarctica, due to its harsh climate, seemed very unlikely to be colonized by annual bluegrass. However, in 1953, specimens of *P*. *annua* were recorded near a ruined whaling station on Deception Island, South Shetland Islands, maritime Antarctica [8]. Since then, it was discovered in several other sites within the region [9]. Its expansion was documented in detail in the vicinity of H. Arctowski Polish Antarctic Station (King George Island, South Shetland Islands), where first sightings of the species were made during the austral summer of 1985–1986, next to the stations living quarters [10,11]. Available historical data point toward Poland-exported soils for greenhouse cultivation purposes as the most likely source of viable *P*. *annua* diaspores and its subsequent introduction [8]. However, molecular analyses indicate a constant inflow of fresh bluegrass genetic material into the introduced population, mostly due to ongoing human activity [2,12]. Primarily occurring in human-disturbed sites associated with the station’s infrastructure, *P*. *annua* has expanded into recently deglaciated glacier forelands, even entering into Antarctic tundra communities [11,13].

There is a consensus within the scientific community about the positive and negative influence of the associated microorganisms on such plant traits as nutrient acquisition, growth, biomass production, and flowering time but also stress tolerance and disease resistance [14,15]. Those multifactorial plant-microbe interactions take place mostly in two major rhizocompartments within the boundaries of the soil-root system mass [16]. The rhizosphere compartment comprises the soil that is in closest proximity to plant roots, where the microbiome is strongly shaped by root exudates and mucilage and contains a metabolically and phylogenetically diverse community [17]. The endosphere, that is, the root interior, hosts a plant-specific microbiome due to selective entry mechanisms and further biochemical exclusion-enrichment processes [18]. In this respect, invasive plant species that thrive in suboptimal conditions are presumed to rely not only on genetic, physiological, and morpho-anatomical adaptations but also on a mutual relationship with the resident microbiome, which enhances their stress tolerance and contributes to the overall plant strength [19].

In this study, we investigated *P*. *annua* root-associated microbiomes sampled in Central Europe (Poland) and maritime Antarctica (King George Island, South Shetland Islands) in terms of bacterial phylogenetic diversity (16S rRNA gene-targeted next-generation amplicon sequencing), metabolic traits (community-level physiological profiling by Biolog Ecoplates) and a geochemical background (soil biogenic component concentrations). The main aim of this investigation was to compare microbial communities residing in the rhizosphere and the root endosphere of *P*. *annua* specimens that grow in their native European settings with those growing in maritime Antarctica as invasive plant species. Our hypothesis states that the differences between microbial communities of Central European and maritime Antarctic *P*. *annua* roots are severe in the rhizospheric compartment yet are minor in the root endosphere, both metabolically and phylogenetically.

This is the first study that sheds light on the microbiome associated with an invasive plant in Antarctica and the first to provide insight into *P*. *annua* associated bacterial community structure by a culture-independent approach.

## 2. Materials and Methods

### 2.1. Sites and Sampling

Antarctic material sampling was carried out in the vicinity of the H. Arctowski Polish Antarctic Station, located at the western shore of Admiralty Bay, King George Island (South Shetlands Islands, maritime Antarctica). Samples were collected during the austral summer season of 2017–2018 from three sites where *P*. *annua* growth was noted. The maximal temperature on the day of sampling was 6.7 °C. Central European material sampling was carried out at the Botanical Garden of the Polish Academy of Sciences (Powsin, city, Poland) in the fall of 2018 (Table 1). The maximal temperature on the day of sampling was 17.3 °C. Several specimens (4–6 per site) were collected with the root-adjacent soil with the use of sterile tools into sterile plastic containers and transported frozen (−20 °C) to the laboratory in the Institute of Biochemistry and Biophysics, Polish Academy of Sciences (IBB PAS). Additionally, bulk soil samples from those sites were gathered in triplicates (approx. 1.5 kg per site) for component analysis and transported in the same conditions.

### 2.2. Bacterial Extraction

Bacterial cells were extracted from fifteen (three per site) individual *P*. *annua* specimen rhizospheric soil and roots. The following method was devised based on the findings of [20] regarding the separation of prokaryotic cells from mineral and organic debris and the guidelines provided by [21] regarding root-associated microbe isolation. To analyze the microbiome of the root-adjacent soil, a sample of the soil was carefully removed from between the roots with a sterile spatula onto a pre-sterilized aluminum foil piece. Approx. 1 g of the soil was weighed and placed in a 50 mL conical tube containing 20 mL of sterile and cool (4 °C) dilution liquid composed of 0.9% (*w/v*) saline (NaCl) and 10 mM tetrasodium pyrophosphate (Na_4_P_2_O_7_). The suspension was then shaken for 30 min in a TornadoTM Vortexer at 2000 rpm at 4 °C. The tubes were then placed in a VWR Ultrasonic Cleaner USC-TH filled with chilled water and sonicated for 60 s. The tubes were vortexed afterward for 30 s to suspend detached cells. After brief centrifugation (1 min; 1000 rpm; 4 °C), the suspension was submitted to metabolic fingerprinting by the Biolog Ecoplate technique. To detach the rest of the adhering soil, the root system was washed in 60 mL of sterile NaCl/Na_4_P_2_O_7_ solution by shaking for 30 min in the aforementioned shaker (1000 rpm; 4 °C) and then rinsed 3 times in 5 mL of the same sterile and cooled solution by vortexing. Washed roots were sterilized by incubation in a cooled 10% hydrogen peroxide (H_2_O_2_) solution for 5 min, then rinsed 3 times with sterile NaCl/Na_4_P_2_O_7_ solution. The so surface-sterilized roots were placed in a pre-cooled sterile mortar. Two and a half ml of sterile NaCl/Na_4_P_2_O_7_ was added with 0.6 g of sterile, sharp garnet sand (lysing matrix A) and gently ground with a pestle, allowing the sharp angular garnet pieces to comminute the roots to an amorphous pulp. The pulp was transferred to a 50 mL conical tube containing 20 mL of sterile and cool (4 °C) NaCl/Na_4_P_2_O_7_ solution and submitted to the above-mentioned procedure (shaking, ultrasonication and vortexing). The resulting supernatant suspension was submitted to metabolic fingerprinting by the Biolog Ecoplate technique and DNA extraction.

### 2.3. DNA Extraction and Targeted 16S rRNA Gene Amplicon Sequencing

Rhizosphere soil DNA was extracted using the PowerSoil^®^ DNA isolation kit (QIAGEN GmbH, Hilden, Germany) according to manufacturer protocol. An approx. 0.2 g of soil was used in triplicates. DNA solutions were kept at 4 °C for further analysis. The dilution liquid containing endosphere bacteria was passed through a sterile 47 mm Whatman polycarbonate filter (0.22 μm pore size). The DNA from the filter-trapped bacteria was extracted using the PowerWater^®^ DNA isolation kit (QIAGEN, GmbH, Hilden, Germany) according to manufacturer protocol and kept at 4 °C. This resulted in 72 DNA samples. The phylogenetic study was performed by targeted sequencing and analysis of the prokaryotic 16S ribosomal RNA gene. A fragment of the 16S rRNA gene containing the V3 and V4 variable regions was amplified using gene-specific primers: 16S_V3-F and 16S_V4-R positions 341-357F and 785-805R, respectively, according to *Escherichia coli* 16S rRNA gene reference sequence [22]. Illumina Nextera XT overhang adapter nucleotide sequences were included in addition to the 16S rRNA gene-specific sequences, which allowed sample indexing and pooling. Each PCR amplification was conducted in triplicates using KAPA HiFi PCR kit (Roche, Basel, Switzerland) in a final volume of 20 μL per reaction according to the manufacturer’s instructions. Obtained PCR products were pooled into 10 samples (2 rhizocompartments × 5 sampling sites) in equimolar ratio and indexed using Nextera XT barcodes (Illumina, San Diego, CA, USA). Amplicon libraries were sequenced on Illumina MiSeq instrument (Illumina, San Diego, CA, USA) in the DNA Sequencing and Oligonucleotide Synthesis Laboratory (IBB, PAS). Sequencing was conducted in paired-end mode (2 × 300 bp) with the use of a v.3 (600 cycles) chemistry cartridge, which allowed the generation of long paired reads fully covering 16S V3–V4 amplicons.

### 2.4. Phenotype Fingerprinting with Biolog EcoPlate™

The EcoPlate Biolog assays assess the ability of a mixed microbial community to use any of 31 carbon compounds as the sole carbon source (plus a single control well with no-carbon). Microbial communities were characterized for their ability to catabolize 10 different carbohydrates, 9 carboxylic and acetic acids, 4 polymers, 6 amino acids, and 2 amines [23]. Root-associated bacterial suspensions were adjusted with sterile 0.9% saline to optical transmittance of 0.9. One hundred microliter aliquots of each suspension were added to each well of EcoPlate microplates (Biolog Inc., Hayward, CA, USA). The plates were incubated in darkness at 10 °C for Antarctic samples and 18 °C for European material. The temperatures were chosen to accommodate the activity range of the respective microbial communities: psychrophiles and psychrotrophes for Antarctic material, psychrotrophes and mesophiles for European material [24]. The color development (absorbance) was read at 590 nm (A_590_) in a Varioscan plate reader (Thermofisher Scientific, Waltham, MA, USA), and cellular respiration was measured kinetically by determining the colorimetric reduction of tetrazolium dye. Data were collected approximately twice a week over a 65 day period. The prolonged incubation of EcoPlates was based on our previous observations [25,26,27]. Data from the forty-second day (Antarctic samples) and twenty-first day (European samples) of incubation were used as there was no further color development after this date. Final absorbance data were first blanked against the time zero reading and then blanked against the respective control well containing no-carbon source. Readings that had the A_590_ value of 0.25 or higher were scored as a positive EcoPlate response (PER).

### 2.5. Measurement of Soil Components

Soil pH (in 1 M KCl) and salinity (in double-distilled water (ddH2O)) were measured with a CPC-411 Elmetron™ multiparameter probe according to [28]. Phosphates and nitrates were determined spectrophotometrically in a Shimadzu UV 1601 spectrophotometer and in an Epoll-Eco 20 spectrophotometer respectively. Other elements were determined by atomic absorption spectroscopy [29].

### 2.6. Data Analysis

Raw sequencing data were cleaned, aligned, and classified automatically by the EzBioCloud platform using the PKSSU4.0 database [30]. Chimeric, low quality, and non-target (chloroplast, mitochondrial, and archaeal) amplicons were automatically discarded. The operational taxonomic unit was defined as a group of sequences that exhibit greater than 97% similarity to each other. Illumina reads were deposited in the NCBI Sequence Read Archive (SRA) as BioProject PRJNA678861. All results were compiled using Excel (MS Office) 2016 for Windows. A two-sample *t*-test was applied to compare different data sets. Variance within the sets was assessed using the f-test beforehand. Correlations between biological and geochemical parameters were calculated using Pearson’s correlation coefficient. Principal component analysis was performed using the singular value decomposition method. Data visualization and statistical analysis has been performed using the R software (R v.4.0.2) and the following packages: ggplot2, fmsb, Hmisc, ggpubr, corrplot, and autoplot [31].

## 3. Results

### 3.1. Soil Components

The nutrient-poor European soil sample (S1) was characterized by high calcium content (5000 mg/100 g) and relatively high pH (7.8), with subsequently low magnesium (38.4 mg/100 g), manganese (52.8 mg/kg), zinc (5.9 mg/kg), copper (2.7 mg/kg), iron and sodium content (4.9 mg/100 g) as well as relatively low nitrate (17.4 mg/100 g), labile phosphorus (6 mg/100 g), and labile potassium (36.1 mg/100 g). The fertilized European soil sample (S2) had very high nitrate, labile phosphorus, and potassium levels (27.5, 219.2, and 251.8 mg per 100 g of soil, respectively). pH in this sample was neutral (7.0) with the highest manganese (218.1 mg/kg) in all examined samples. Antarctic samples were characterized by overall low nitrate (7.7–16.3 mg/100 g), labile phosphorus (4.9–15.1 mg/100 g), and potassium concentration (31.1–55.8 mg/100 g) values, high salinity (0.4 g NaCl/L), and high magnesium (205–250.7 mg/100 g) and iron contents (4269.8–4670.2 mg/kg). Zinc was low in the glacial foreland sample (9.2 mg/kg) (Table 2).

### 3.2. Diversity Indices

**Figure 1:**
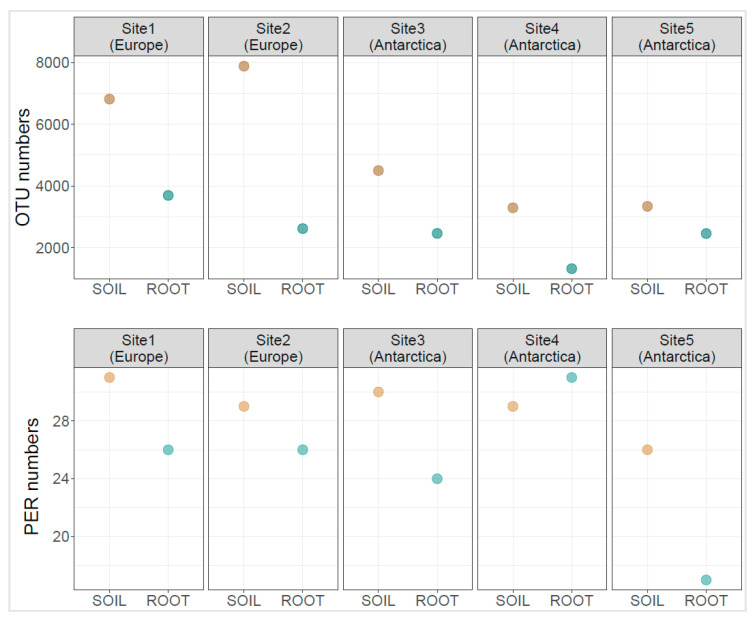
Operational taxonomic unit (OTU) numbers (upper graphs) and positive EcoPlate response (PER) numbers (lower graphs) for the bacterial communities associated with *Poa annua* roots. OTU numbers represent a value derived from three pooled samples; positive EcoPlate responses are the mean value of 9 replicates.

The highest diversity as assessed by operational taxonomic unit numbers (OTUs) were noted for the fertile Central European soil sample P2S (OTU = 7879), followed by Central European soil sample P1S (OTU = 6813). Antarctic soil samples displayed lower bacterial phylogenetic diversity. Sample P3S showed the highest diversity among them (OTU = 4498), whereas the proglacial soil sample P4S the lowest diversity (OTU = 1322). Bacterial communities residing within the roots displayed lower diversity (av. 2512.4 OTU, sd. 841) than the corresponding rhizosphere soil (av. 5165 OTU, sd. 2083). The most diverse was the bacterial community in soil sample P1R from grass specimens growing in nutrient-poor European soil (OTU = 3694). Samples P2R showed lower values (OTU = 2620). Root-residing bacteriome was diverse in plants growing near the Arctowski Antarctic station: P3R (OTU = 2465) and P5R (OTU = 2461), the one residing in plants growing in postglacial soil (P4R) showed relatively low diversity (OTU = 1322). Other diversity indices are presented in Supplementary File S1. The number of positive EcoPlate responses in rhizospheric soil samples varied between 26 (sample P5S) and 31 (sample P1S), whereas for the root samples, it varied between 17 (sample P5R) and 31 (sample P4R) (Figure 1).

### 3.3. Bacterial Phylogenetic Diversity

**Figure 2:**
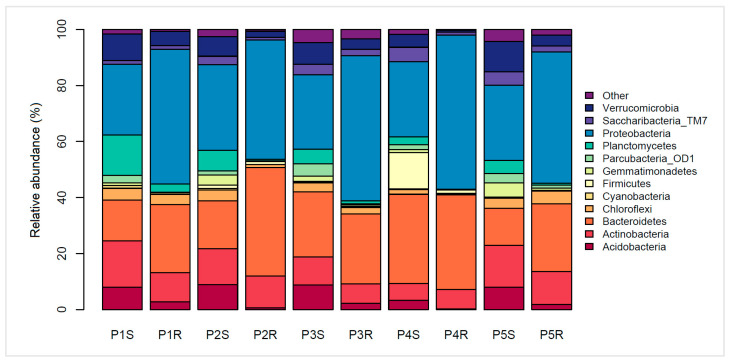
Relative abundance by percentile contribution of sequences identified on a phylum-rank taxonomic level. Other—group of bacterial phyla below 1% relative abundance each, S—rhizospheric soil samples, R—root samples, P1–P2—Central European (Poland) samples, P3–P5—Antarctic samples (King George Island).

Twelve major (>1%) phyla were found in the rhizosphere and root interior of the *P*. *annua* specimens (Figure 2). Proteobacterial sequences were the most numerous in investigated samples. According to their percentage contribution, Proteobacteria have been enriched in the root-interior compartment in comparison to the respective rhizosphere soil community. The enrichment is approx. 2-fold in every case analyzed, being it European (P1S/P1R = 25.32%/48.14%; P2S/P2R = 30.5%/42.52%) or Antarctic material (P3S/P3R = 26.60%/51.83%; P4S/P4R = 26.80%/55.04%; P5S/P5R = 26.79%/46.87%). Sequences belonging to the Bacteroidetes phylum also were highly abundant in the sampling material. Their enrichment in the root interior was apparent only in some cases (P1S/P1R = 14.53%/24.32%; P2S/P2R = 17.04%/38.68%; P5S/P5R = 13.18%/24.12%), whereas not so in others (P3S/P3R = 23.18%/24.96%; P4S/P4R = 31.90%/33.84%). Acidobacterial presence was reduced in root interior compared to the rhizosphere soil (P1S/P1R = 8.01%/2.77%; P2S/P2R = 8.95%/0.65%; P3S/P3R = 8.72%/2.25%; P4S/P4R = 3.29%/0.19%; P5S/P5R = 7.98%/1.81%). Similarly, Planctomycetes abundance also diminished inside roots (P1S/P1R = 14.38%/2.90%; P2S/P2R = 7.39%/0.61%; P3S/P3R = 5.28%/1.09%; P4S/P4R = 2.92%/0.18%; P5S/P5R = 4.67%/0.65%). Noteworthy are the low Verrucomicrobia numbers in the root interior of specimens growing in postglacial soils (P4R = 0.71%) and high Firmicutes numbers in the corresponding rhizosphere soil (P4S = 13%).

**Figure 3:**
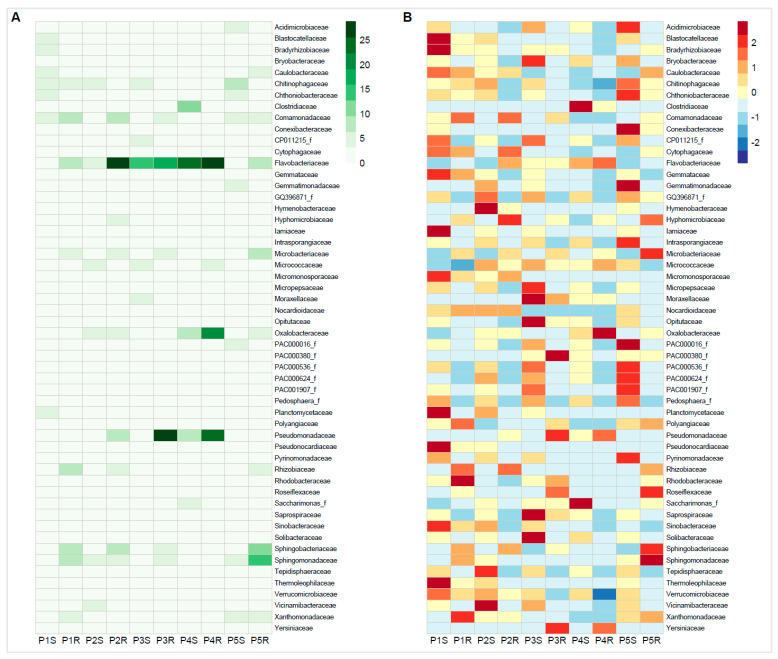
Relative abundance heatmap of sequences identified on a family-rank taxonomic level. (**A**)—Relative abundance according to sequence percentage value; color scale—relative abundance (%), (**B**)—Scaling within family rows across all examined samples; relative abundance above average—red, relative abundance below average—blue. S—rhizospheric soil samples, R—root samples, P1–P2—Central European (Poland) samples, P3–P5—Antarctic samples (King George Island).

Relative abundance of family-rank groups revealed several discrepancies, but also similarities between European and Antarctic *P*. *annua* root-associated bacterial communities (Figure 3, Supplementary File S2). In the rhizosphere community, the most noticeable was the difference in the relative abundances of the family *Flavobacteriaceae* (Europe—av. 3.6%; Antarctica—av. 13.9%) and also of the *Planctomycetaceae* (Europe—av. 4.5%; Antarctica—av. 1.1%). Some families displayed a comparable average relative abundance in both sample types, most notably the *Chitinophagaceae* (Europe—av. 4.5%; Antarctica—av. 4.1%) and the *Chthoniobacteraceae* (Europe—av. 3.0%; Antarctica—av. 2.9%). High relative abundance in the rhizosphere of a few families was site-specific. Site 4 (proglacial terrain) was especially rich in the following: *Clostridiaceae* (12.7%), *Oxalobacteraceae* (8.3%), and *Pseudomonadaceae* (7.4%). The endosphere community harbored high relative abundances of the family *Flavobacteriaceae*, both for the European (av. 17.5%) and the Antarctic (av. 17.7%) material. Major differences between European and Antarctic root-endosphere communities could be noticed in the relatively low abundances for the latter of the families: *Rhizobiaceae* (Europe—av. 6.6%; Antarctica—av. 2.4%) and *Comamonadaceae* (Europe—av. 7.3%; Antarctica—av. 3.3%). Similar to the rhizosphere, some endospheric families displayed unusually high relative abundances only at specific sites. Site 3 was particularly rich in *Pseudomonadaceae* sequences (25.8%), Site 4 in *Oxalobacteraceae* sequences (21.0%), and Site 5 in *Sphingomonadaceae* (15.3%) and *Sphingobacteriaceae* sequences (10.2%).

### 3.4. Community-Level Physiological Profiling by Biolog EcoPlates

**Figure 4:**
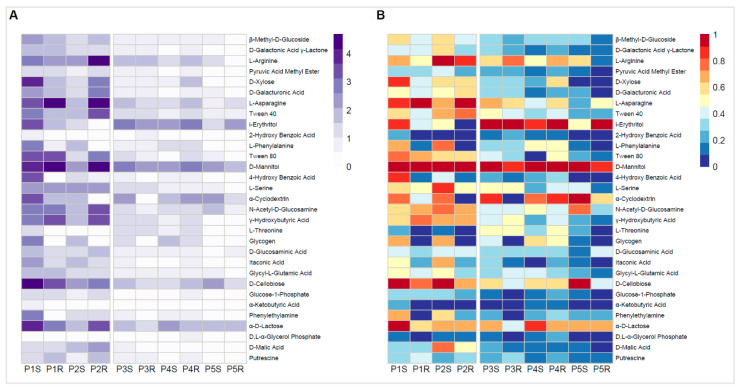
Heatmap displaying *Poa annua* rhizosphere and root community responses on Biolog Ecoplates. (**A**)—mean absorbance values from three replicates; color scale—absorbance values at 590 nm, (**B**)—scaling within a particular carbon source across all examined samples; absorbance above average—red, absorbance below average—blue. S—rhizospheric soil samples, R—root samples, P1–P2—Central European (Poland) samples, P3–P5—Antarctic samples (King George Island).

Microbial community response intensity in Biolog Ecoplates was considerably higher for the European samples than in those obtained from Antarctic material (Figure 4, Supplementary File S3). In order to be comparable, the data needed to be normalized in terms of intensity value (values within a particular sample type were divided by the highest value in that sample). The highest absorbance value at 590 nm (A_590_) obtained in the European material was A_590_ = 4.69, whereas, for the Antarctic samples, A_590_ = 2.82. D-mannitol was the most intensely metabolized carbon source by all examined microbial communities, especially by root-inhabiting communities from European material (A_590_ = 4.49–4.69). Apart from that, severe discrepancies in the metabolism of other compounds have been observed. Microbial community residing in nutrient-poor rhizospheric soil (P1S) was most efficient in metabolizing D-cellobiose (A_590_ = 4.27) and α-D-lactose (A_590_ = 4.03) as well as D-xylose (A_590_ = 3.81), L-asparagine (A_590_ = 3.59), i-erythritol (A_590_ = 3.60), and 4-hydroxy benzoic acid (A_590_ = 3.52). The corresponding root community (P1R) efficiently metabolized L-asparagine (A_590_ = 4.27), γ-hydroxybutyric acid (A_590_ = 3.57), and also D-cellobiose (A_590_ = 3.33). The fertile European rhizospheric soil community (P2S) provided the strongest responses to L-arginine (A_590_ = 2.44), L-serine (A_590_ = 2.27), L-phenylalanine (A_590_ = 1.98), α-cyclodextrin (A_590_ = 1.97), N-acetyl-D-glucosamine (A_590_ = 2.05), D-cellobiose (A_590_ = 2.44), and D-malic acid (A_590_ = 1.94). The corresponding root community metabolized preferably: L-arginine (A_590_ = 4.21) and L-asparagine (A_590_ = 4.62) but also Tween40 (A_590_ = 3.45). Apart from metabolizing D-mannitol, the Antarctic rhizosphere and root communities had an affinity for i-erythritol (A_590_ = 2.30–2.82), except for the P5S sample (A_590_ = 1.18). α-cyclodextrin and α-D-lactose, except P3R in both cases, was also readily metabolized by the Antarctic microbial community (A_590_ = 1.38–2.46 and A_590_ = 1.57–2.30, respectively). Efficient N-acetyl-D-glucosamine and D-cellobiose metabolism were apparent only in sample P5S (A_590_ = 1.83 and A_590_ = 2.44, respectively).

### 3.5. Correlations between Biological and Geochemical Data

**Figure 5:**
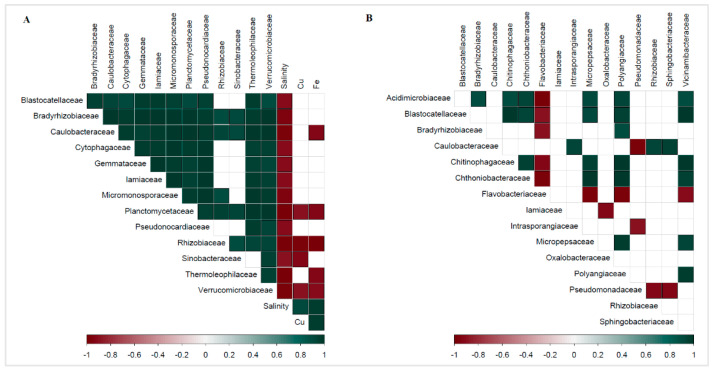
(**A**)—Correlogram of rhizospheric family-rank sequence abundance data and soil chemistry. Only significant (*p* < 0.05) correlations are shown. (**B**)—Correlogram of root endosphere family-rank sequence abundance data. Only significant (*p* < 0.05) correlations are shown; the color scale represents correlation coefficient values: dark green—positive correlation, dark red—negative correlation.

Pearson’s correlation coefficient between the percentile abundance of bacterial families within the rhizosphere and the chemical composition of the soil revealed several significant (*p* < 0.05) negative correlations (Figure 5A, Supplementary File S4). Salinity displayed the highest number of negative correlations with bacterial family-rank groups: *Thermoleophilaceae* (r = −0.98, *p* = 0.002), *Rhizobiaceae* (r = −0.98, *p* = 0.003), *Planctomycetaceae* (r = −0.98, *p* = 0.003), *Caulobacteraceae* (r = −0.98, *p* = 0.004), *Verrucomicrobiaceae* (r = −0.96, *p* = 0.01), *Micromonosporaceae* (r = −0.95, *p* = 0.01), *Bradyrhizobiaceae* (−0.95, *p* = 0.01), *Pseudonocardiaceae* (r = −0.92, *p* = 0.03), *Iamiaceae* (r = −0.92, *p* = 0.03), *Cytophagaceae* (r = −0.91, *p* = 0.03), *Blastocatellaceae* (r = −0.90, *p* = 0.04), *Gemmataceae* (r =−0.90, *p* = 0.04), and *Sinobacteraceae* (r = −0.89, *p* = 0.04). Iron concentration showed negative correlations with the relative abundance of the following families: *Rhizobiaceae* (r = −0.99, *p* = 0.001), *Thermoleophilaceae* (r = −0.94, *p* = 0.02), *Planctomycetaceae* (r = −0.93, *p* = 0.02), *Caulobacteraceae* (r = −0.92, *p* = 0.03), and *Verrucomicrobiaceae* (r = −0.91, *p* = 0.03). Copper concentrations negatively correlated with the relative abundance of the following bacterial families: *Rhizobiaceae* (r = −0.96, *p* = 0.01), *Sinobacteraceae* (r = −0.93, *p* = 0.02), *Verrucomicrobiaceae* (r = −0.90, *p* = 0.04), and *Planctomycetaceae* (r = −0.89, *p* = 0.04). Pearson’s correlation coefficient between the percentile abundance of bacterial families of the *P*. *annua* root endosphere highlighted several significant correlations (Figure 5B, Supplementary File S4). Most were obtained for the *Flavobacteriaceae*, which were negatively correlated with: *Acidimicrobiaceae* (r = −0.99, *p* = 0.002), *Chthoniobacteraceae* (r = −0.98, *p* = 0.004), *Micropepsaceae* (r = −0.97, *p* = 0.01), *Polyangiaceae* (r = −0.96, *p* = 0.01), *Chitinophagaceae* (r = −0.92, *p* = 0.03), *Vicinamibacteraceae* (r = −0.92, *p* = 0.03), *Blastocatellaceae* (r = −0.89, *p* = 0.04), and *Bradyrhizobiaceae* (r = −0.89, *p* = 0.04). The *Pseudomonadaceae* abundance displayed negative correlations with the contribution of: *Caulobacteraceae* (r = −0.97, *p* = 0.01), *Rhizobiaceae* (r = −0.94, *p* = 0.02), *Sphingobacteriaceae* (r = −0.93, *p* = 0.02), and *Intrasporangiaceae* (r = −0.90, *p* = 0.04). The *Oxalobacteraceae* displayed a negative correlation with the *Iamiaceae* (r = −0.93, *p* = 0.02).

### 3.6. Principal Component Analysis

**Figure 6:**
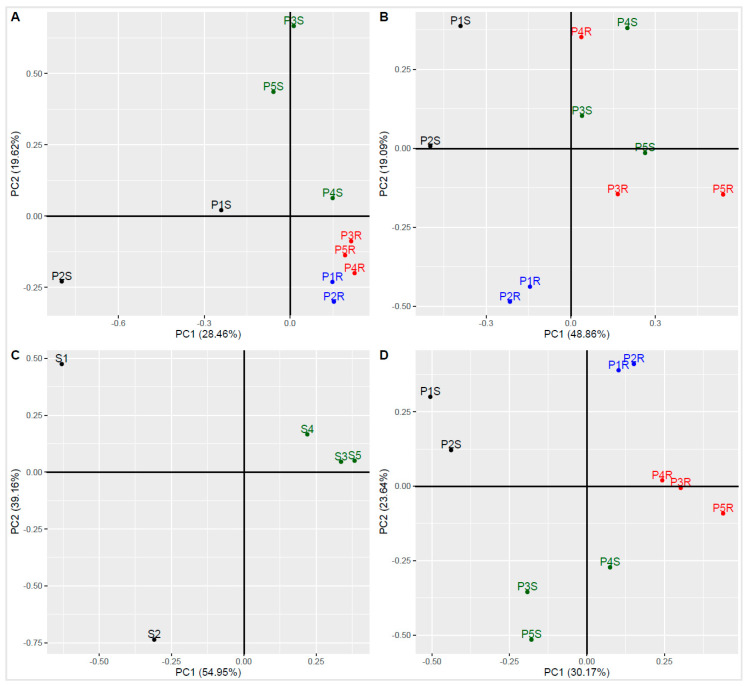
Principal component analysis (PCA) of biological and chemical data. (**A**)—PCA based on percentage contribution of bacterial sequences identified on a family-rank level. (**B**)—PCA based on normalized responses obtained for bacterial communities by the Biolog Ecoplate method. (**C**)—PCA based on soil chemical data. (**D**)—PCA based on a combination of family-rank bacterial sequence percentile contribution and normalized community responses on Biolog Ecoplates.

Principal component analysis (PCA) based on the abundance of sequences of a family-rank taxon showed a tight clustering of the root-dwelling bacterial communities, both of European and Antarctic origin (Figure 6A). Soil bacterial communities were vastly different, with the proglacial sample P4S bearing the closest resemblance to root communities. PCA based on the Biolog Ecoplate responses showed that European root-interior microbial communities were the most similar despite the differences in their corresponding rhizospheric communities. Antarctic root communities bore more resemblance to their respective rhizosphere communities than to each other or the corresponding European samples (Figure 6B). PCA of soil chemistry data showed a great discrepancy between the nutrient-poor (P1S) and the nutrient-rich European rhizospheric soils (P2S). Antarctic samples clustered closely together, showing great similarity (Figure 6C). PCA clustering made using the combined phylogenetic and functional (Biolog Ecoplate) data revealed four distinct groups: European rhizospheric soil community (P1S, P2S), Antarctic rhizospheric soil community (P3S, P4S, P5S), European root community (P1R, P2R), and Antarctic root community (P3R, P4R, P5R) (Figure 6D).

### 3.7. Significant Differences between Microbial Parameters

**Figure 7:**
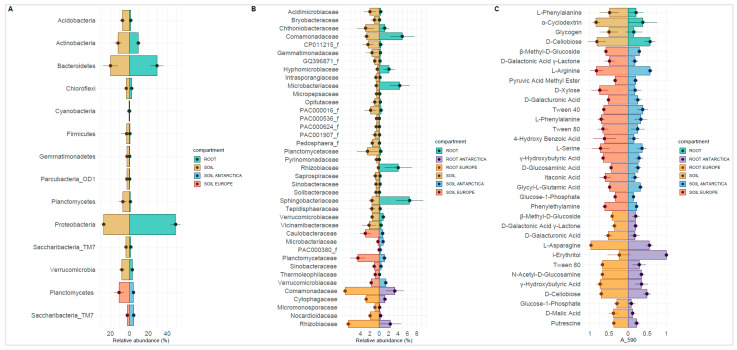
Statistically significant differences (*p* < 0.05) between different groups (see legends) of *Poa annua* root-associated communities: (**A**)—sequence contribution identified on a phylum taxonomic level; (**B**)—sequence contribution identified on a family-rank taxonomic level; (**C**)—community responses on Biolog Ecoplates based on absorbance values at 590 nm (A_590).

The *t*-test calculations showcased significant (*p* < 0.05) differences between Central European and Antarctic rhizospheric microbial parameters (Figure 7). The relative abundance of the following taxa was higher in the European samples: Planctomycetes, *Caulobacteraceae*, *Planctomycetaceae*, *Rhizobiaceae*, *Sinobacteraceae*, *Thermoleophilaceae*, and *Verrucomicrobiaceae*. The catabolic intensity of several compounds was also higher in the Central European rhizosphere samples, most notably for: β-methyl-D-glucoside, phenylethylamine, glucose-1-phosphate, γ-hydroxybutyric acid, itaconic acid, D-galacturonic acid, and D-xylose. Antarctic material had a significantly higher sequence abundance of the Saccharibacteria phylum and the actinobacterial family of *Microbacteriaceae*. Significant differences between the root endosphere communities highlighted higher abundances/intensities of several features in the Central European samples. In those samples, a higher relative abundance of the following bacterial families were noted: *Comamonadaceae*, *Cytophagaceae*, *Micromonosporaceae*, *Nocardioidaceae,* and *Rhizobiaceae*. As for compound use intensity, it was true for the following substrates: β-methyl-D-glucoside, D-galactonic acid γ-lactone, D-galacturonic acid, L-asparagine, Tween 80, N-acetyl-D-glucosamine, γ-hydroxybutyric acid, D-cellobiose, glucose-1-phosphate, D-malic acid, and putrescine. The only feature significantly higher in the Antarctic material was i-erythritol use intensity. Most of the significant differences were apparent between the rhizosphere and the endosphere of *P*. *annua*. The root interior showed significant enrichment in the relative abundance of the following taxa: Proteobacteria, *Microbacteriaceae*, *Sphingobacteriaceae*, *Hyphomicrobiaceae*, *Rhizobiaceae*, Bacteroidetes, and *Comamonadaceae* while also displaying significantly lower catabolism intensities of L-phenylalanine, α-cyclodextrin, glycogen, and D-cellobiose.

### 3.8. Poa annua Core Microbiome

**Figure 8:**
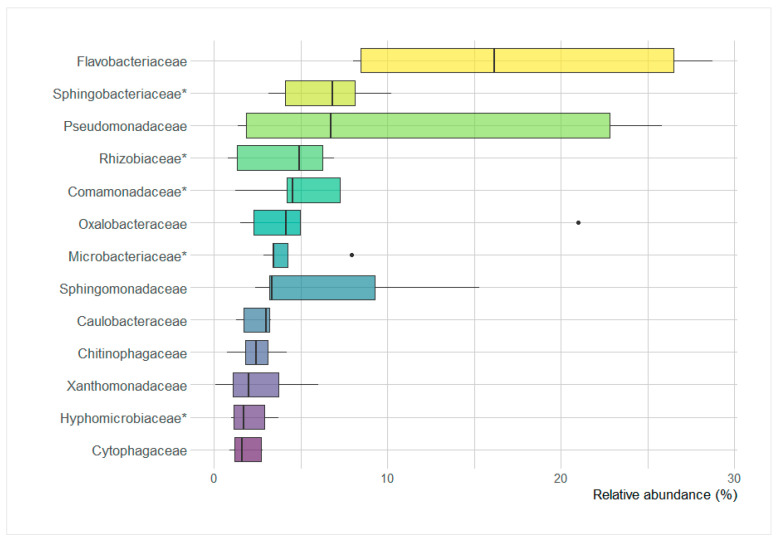
Boxplots displaying the range of relative sequence abundance of major *Poa annua* root-dwelling bacterial families. * significantly enriched in the endosphere compared to the rhizosphere.

Thirteen bacterial families had an average relative abundance higher than the average abundance of family-rank group in the root endosphere and are therefore considered to be the core microbiome taxa of *P*. *annua* in this study (Figure 8). The *Flavobacteriaceae* had the highest abundance on average (8.0–28.7%, Me = 16.2) followed by *Sphingobacteriaceae* (3.1–10.2%, Me = 6.8%), *Pseudomonadaceae* (1.4–22.8%, Me = 6.7%), *Rhizobiaceae* (0.8–6.9%, Me = 4.9%), *Comamonadaceae* (1.2–7.3%, Me = 4.6%), *Oxalobacteraceae* (1.5–21.0%, Me = 4.1%), *Microbacteriaceae* (2.8–7.9%, Me = 3.5%), *Sphingomonadaceae* (2.4–15.3%, Me = 3.3%), *Caulobacteraceae* (1.3–3.3%, Me = 3.0%), *Chitinophagaceae* (0.7–4.2%, Me = 2.4%), *Xanthomonadaceae* (0.1–6.0%, Me = 2.0%), *Hyphomicrobiaceae* (1.0–3.7%, Me = 1.7%), and *Cytophagaceae* (0.9–2.8%, Me = 1.6%).

## 4. Discussion

The multiphasic approach applied in this study revealed several phenomena within the *P*. *annua* root-associated microbiome.

Rhizospheric soils examined in this study each contained a unique bacterial community in terms of phylogenetic diversity, as shown in the Principal Component Analysis. This diversity was higher in the European rhizospheric soil samples than in the Antarctic ones. Antarctic ecosystems were repeatedly proven to be less diverse and less complex than those in lower latitudes [32,33,34]. Geographical isolation was often made responsible for the low diversity of Antarctic macro-organisms [35,36]. However, this kind of isolation may not directly apply to bacteria, as it was discovered that live bacterial cells can be transported over long geographic distances [37]. Regarding rhizospheric microbial communities, their diversity can be largely controlled by host diversity and abundance [38], which in the case of Antarctic flora is indeed limited due to geographic isolation but also due to harsh climatic conditions [39]. The specific geochemical characteristics of the examined Antarctic soils may be even more responsible for the low bacterial diversity [40]. Those soils shared some features such as high salinity, sodium, magnesium, and heavy metal concentrations. This is mainly due to sea spray influence and the volcanic history of this site [41]. These characteristics, especially salinity, showed significant, negative correlations with members belonging to several soil community bacterial families, including the plant growth-promoting *Rhizobiaceae*. Salt heavy metal sensitivity, especially that of free rhizobial cells, has been extensively studied, as it impacts the biomass production of several agricultural plants [42,43,44]. Although there were no significant differences in the relative sequence abundance of the majority of bacterial phyla between European and Antarctic rhizospheric soils, they did significantly differ in the abundance of Planctomycetes and Saccharibacteria. European rhizosphere had a higher contribution of Planctomycetes, members of which are connected to plant biomass decomposition, but also to high soil calcium concentrations, which was noted in those soils [45]. Furthermore, European samples displayed significantly higher contributions of key taxa, such as the *Rhizobiaceae*, a family that contains plant-beneficial nitrogen fixers, and the *Sinobacteraceae*-harboring rhizospheric ammonia oxidizers [46,47]. The members of other families such as *Caulobacteraceae* and *Verrucomicrobiaceae,* known for grass-related rhizospheric competence, have also been found in significantly higher amounts in those samples [48,49]. Antarctic rhizospheric soils had on average a higher contribution of Saccharibacteria sequences. Based on a limited amount of reports about the members of this phylum, they can be suspected to be scavengers or even parasites rather than active biomass degraders [50]. Members of the *Microbacteriaceae* displayed significantly higher amounts in the *P*. *annua* rhizosphere growing in the Antarctic. Rhizospheric enrichment in *Microbacteriaceae* has rarely been observed but could be influenced by climatic factors rather than soil composition [51]. A curious anomaly was noted in the Antarctic rhizospheric soils of postglacial origin. The postglacial rhizospheric soil sample contained high numbers of sequences belonging to the Firmicutes phylum, the majority of which were identified as *Clostridiaceae*, a family containing anaerobic, endospore-forming bacteria [52]. This can be connected to the recent deglaciation event [53]. Subglacial habitats have been speculated to be mostly anoxic. The experiment of [54] confirmed that in such conditions, members of the *Clostridiaceae* could be considerably enriched from Antarctic, supraglacial materials. Furthermore, the spores of the *Clostridiaceae* can persist in Antarctic settings even after the conditions have changed to oxic [26]. Besides higher phylogenetical diversity, European rhizospheric communities also displayed significantly higher use abilities for several types of compounds, such as carbohydrates, amino and carboxylic acids, lipids, and amines. Together with the high phylogenetic diversity, this suggests a plethora of different bacterial niches and high assemblage complexity, hinting at the presence of a rich reservoir of rhizosphere-competent bacteria [55,56].

The rhizosphere is considered the direct reservoir of root endophytes, albeit a selective entry mechanism exists and is well-described [57,58]. In this respect, the phylogenetic diversity in the endosphere was considerably lower than that in the rhizosphere. Furthermore, rhizosphere diversity had a direct impact on the endosphere diversity. This seems to be a staple when examining plant-associated bacterial communities as it was observed for wild plants [59] as well as a field [60] or greenhouse cultivars [61]. The endospheric communities of *P*. *annua* assessed in this study showed much phylogenetic similarity to each other when examined collectively with the rhizosphere community. This was probably due to the depletion in the root of several major soil-dwelling high-ranking taxa such as Acidobacteria and Planctomycetes, along with some other low abundance phyla. Sequences of the Proteobacteria and Bacteroidetes were significantly enriched in the endosphere. Despite this apparent similarity, several differences in the composition of the European and Antarctic endophytic communities could be noticed. Members of the family *Rhizobiaceae* and also *Comamonadaceae* were detected to largely contribute to European root endospheric bacteriomes but were significantly less numerous in Antarctic material. Those families hold nitrogen-fixing members, and their presence can largely increase plant biomass production [62,63]. In addition, the endosphere, as well as the rhizosphere of Antarctic *P*. *annua* specimens, were low in some of the most pivotal actinobacterial taxa found in well-developed soils in lower latitudes, namely the mycelium forming members of the families *Nocardioidaceae* and *Micromonosporaceae*, known for their antimicrobial activities [64,65]. On a family-rank level, there were no significantly enriched groups in the Antarctic endosphere. However, a few bacterial families showed abnormally high contribution in the Antarctic samples, namely: *Oxalobacteraceae*, *Pseudomonadaceae,* and *Sphingomonadaceae*. This is consistent with the findings of [66], where *Pseudomonadaceae* and *Sphingomonadaceae* were among high abundance taxa in the endosphere of native Antarctic plants: *Colobanthus quitensis* and *Deschampsia antarctica*. However, in those communities, the family *Enterobacteriaceae* was present in considerable amounts, unlike in the endosphere of examined here *P*. *annua*. The *Pseudomonadaceae* showed negative correlations with several key plant endophytic taxa such as the *Rhizobiaceae*, *Intrasporangiaceae*, *Caulobacteraceae,* and *Sphingobacteriaceae*, whereas the *Oxalobacteraceae* were negatively correlated with the *Iamiaceae*. Microbial interactions are usually very complex, albeit, within the root interior, they are more simplified due to the selection-mediated diversity impoverishment. In this respect, this could be a case of antagonisms or niche overlap. A recent study on the endophytic *Oxalobacteraceae* member *Massilia* sp. revealed its copiotrophic and r-strategy based lifestyle while also being competition-sensitive [67]. Such an explanation could be plausible in the Antarctic habitat, especially in the low diversity proglacial site, which houses opportunistic pioneer species [68]. The principal component analysis of the catabolic responses showed an interesting feature of the Antarctic bacterial endophytic community. While the Central European endophytic communities differed from the corresponding rhizospheric communities (and being more metabolically diverse), the Antarctic endophyte communities showed more similarity to the matching rhizospheric communities. This points toward Antarctic soil bacterial communities being catabolically fixed on certain abundant and stable nutrient sources, and the root-incorporated bacteria might not be true endophyte specialists. Furthermore, i-erythritol catabolism intensity was significantly higher in the Antarctic endophytic community. This sugar alcohol, contrary to the popular Mannitol, is not produced by plants but by green algae [69]. Antarctic soils house different species of aeroterrestrial algae, such as the widespread in the Antarctic region *Prasiola crispa* [10]. This compound’s high catabolism potential in the Antarctic *P*. *annua* roots could mean aeroterrestrial algae-associated bacteria incorporation into the endophytic community. Despite the aforementioned dissimilarities between root communities, a core bacterial endophytobiome of *P*. *annua* can be extrapolated. The family *Flavobacteriaceae* has been on average the most abundant in the root interior, despite a wide abundance range. Only in recent years have the members of this family been recognized as important contributors to the root endosphere community [70] and were found to largely contribute to the endosphere of Antarctic native grass *D*. *antarctica* [71]. It is suspected that a metabolically diverse pool of *Flavobacterium* spp. is constantly present in the root interior, shifting in abundance patterns in response to environmental conditions and the physiological state of the host plant [70]. The latter is supported by the findings of [72], where plant-associated factors dictated flavobacterial presence. Investigations on temperate region invasive plants’ root microbiota indicate that they increase the fitness of the host in non-native settings mainly by enhancing the acquisition of nutrients such as phosphorous and nitrogen [73]. However, in polar regions, the alleviation of cold-induced stresses may be of key importance for alien plant establishment [74]. In this respect, studies on the bacterially mediated cold resistance of agricultural crops point toward the action of ACC deaminase [75,76]. This microbially produced enzyme lowers the concentration of plant stress hormone ethylene, a substance that inflicts a significant reduction in plant growth and development [77]. Certain strains of the genus *Flavobacterium* have displayed ACC deaminase activity [78]. In consequence, *Flavobacteriaceae* bacteria may play a crucial role in *P*. *annua* adaptation to Antarctic conditions by modifying stress responses of the plant during adaptation [77].

## 5. Conclusions

In conclusion, bacterial root-associated communities of *P*. *annua* differed both phylogenetically and metabolically between those of Central European and those of maritime Antarctic origin. Several key plant-beneficial bacterial groups were less abundant in the Antarctic material, probably due to the geochemical makeup of the soil. Some bacterial families displayed unusually high abundance in the root endosphere of the Antarctic *P*. *annua* specimens. They most likely contained competition-sensitive opportunists that proliferated in the absence of antagonistic microbes. Nonetheless, an endophytic core microbiome could be assumed consisting of 13 bacterial families belonging to the Proteobacteria, Bacteroidetes, and Actinobacteria phyla. The *Flavobacteriaceae* family was the most numerous and most likely to positively influence the adaptation of *P*. *annua* to Antarctic conditions.

## Figures and Tables

**Table 1:** Sampling site characteristics; m. a. s. l.—meters above sea level.

Sampling Site	Geographical Coordinates	Distance to the Sea	Altitude	Structure of Vegetation	Landform and Habitat
P1	52°06′22″ N 21°05′56″ E Powsin, Poland	285 km	90 m.a.s.l.	*Poa annua* (90%) and *Poa pratensis* (10%).	Rubble path, sand, and sandstone. Dry site, sheltered away from sources of nutrients.
P2	52°06′29″ N 21°05′33″ E Powsin, Poland	285 km	95 m.a.s.l.	*Poa annua* (30%), *Malus domestica* (10%), *Trifolium arvense* (30%), *Sonchus oleraceus* L. (10%), *Sonchus arvensis* (20%).	Soil, mechanically altered by human activities. Wet site, fertile, extra fertilization once a year with an animal post-food mass.
P3	62°09′35″ S 58°28′26″ W Arctowski Station, Antarctica	120 m	0.5 m.a.s.l.,	*Colobanthus quitensis* (15%), *Deschampsia antarctica* (25%), *Poa annua* (10%), mosses (25%) and fruticose, foliose and crustose lichens (25%).	Base station, soil mechanically altered by human activities; Skeletic Eutric Fluvisol (Turbic) The site is strongly influenced by marine aerosols, moist, with large human influence.
P4	62°10′05″ S 58°27′46″ W Ecology Glacier foreland, Antarctica	20 m	0.5 m.a.s.l.,	Mosses (40%), fruticose and foliose lichens (40%), *Colobanthus quitensis* (10%) and *Deschampsia antarctica* (10%).	Fluted moraine; Eutric Skeletic Protic Regosol (Turbic). Dry site, sheltered away from sources of nutrients with a little influence of marine aerosols.
P5	62°09′33″ S 58°28′25″ W Arctowski Station, Antarctica	100 m	0.5 m.a.s.l.,	*Colobanthus quitensis* (15%), *Deschampsia antarctica* (25%), *Poa annua* (10%), mosses (25%) and fruticose, foliose and crustose lichens (25%).	Base station, soil, mechanically altered by human activities; Skeletic Eutric Fluvisol (Turbic) The site is medium influenced by marine aerosols, moist, with large human influence, especially by using big vehicles.

**Table 2:** Soil component concentrations.

Soil Components	S1	S2	S3	S4	S5
N (mgNO_3_/100 g)	17.4	27.5	8.8	16.3	7.7
P (mgP_2_O_5_/100 g)	6.0	219.2	5.6	15.1	4.9
K (mg K_2_O/100 g)	36.1	252.8	31.1	55.8	31.9
Mg (mg Mg/100 g)	38.4	185.4	244.7	205	250.7
Ca (mg Ca/100 g)	>5000	2024.6	498.0	1020.1	533.3
Na (mg Na/100 g)	4.9	4.0	>150	>150	>150
Salinity (g NaCl/L)	0.2	0.3	0.4	0.4	0.4
pH	7.8	7.0	7.4	7.5	7.4
Mn (mg Mn/kg)	52.8	218.1	147.5	132.1	155.5
Zn (mg Zn/kg)	5.9	>50	>50	9.2	>50
Cu (mg Cu/kg)	2.7	5.7	21.8	>30	29.8
Fe (mg Fe/kg)	389.4	1502.5	4412.6	4670.2	4269.8

N—nitrate nitrogen, P—labile phosphorus, K—labile potassium, S1–S2—European samples, S3–S5—Antarctic samples.

## Data Availability

Illumina reads were deposited in the NCBI Sequence Read Archive (SRA) as BioProject PRJNA678861.

## Supplementary Materials

The following are available online at https://www.mdpi.com/article/10.3390/microorganisms9040811/s1, Table S1: Alpha-diversity indices for the bacterial communities associated with *Poa annua* roots, Table S2: Relative abundance heatmap of sequences identified on a family-rank taxonomic level, Table S3: Heatmap displaying *Poa annua* rhizosphere and root community responses on Biolog Ecoplates, Table S4: Correlations of rhizospheric family-rank sequence abundance data and soil chemistry, Table S5: Correlations between root endosphere family-rank sequence abundance data.
