# Supplementary material for: Bacterial Communities Associated with Poa annua Roots in Central European (Poland) and Antarctic Settings (King George Island)
Source: Microorganisms. 2021 Apr 12;9(4):811. doi: 10.3390/microorganisms9040811 (PMC8069831; doi:10.3390/microorganisms9040811)
Supplement: Supplementary file 1 [file microorganisms-09-00811-s001.zip › microorganisms-1161628-supplementary/Supplementary file 1.docx]

| **Sample Name** | **Target Reads** | **OTUs** | **ACE** | **CHAO** | **Jackknife** | **NPShannon** | **Shannon** | **Phylogenetic Diversity** |
| --- | --- | --- | --- | --- | --- | --- | --- | --- |
| **P1S** | 70766 | 6813 | 7414.08 | 7146.76 | 7855 | 7.88 | 7.78 | 6422 |
| **P1R** | 75319 | 3694 | 4261.12 | 4077.01 | 4456 | 6.52 | 6.46 | 4326 |
| **P2S** | 75138 | 7879 | 8867.08 | 8488.36 | 9400 | 7.89 | 7.77 | 8195 |
| **P2R** | 56144 | 2620 | 3579.53 | 3398.97 | 3895.73 | 5.71 | 5.64 | 3701 |
| **P3S** | 74453 | 4498 | 4856.76 | 4697.63 | 5131 | 6.83 | 6.76 | 4669 |
| **P3R** | 75684 | 2465 | 2822.62 | 2683.62 | 2955 | 5.08 | 5.03 | 3113 |
| **P4S** | 77524 | 3295 | 3645.49 | 3491.67 | 3854 | 5.49 | 5.43 | 3829 |
| **P4R** | 76608 | 1322 | 1682.99 | 1604.25 | 1715.91 | 4.13 | 4.1 | 1969 |
| **P5S** | 30997 | 3340 | 3838.85 | 3674.42 | 4053 | 7.1 | 6.99 | 4054 |
| **P5R** | 67373 | 2461 | 2851.85 | 2735.1 | 2984 | 5.65 | 5.61 | 3294 |

**Table S1.** Alpha-diversity indices for the bacterial communities associated with *Poa annua* roots. P1–P2 – European samples, P3-P5 – Antarctic samples, S – rhizospheric soil, R – root interior.
