# Supplementary material for: Bacterial Communities Associated with Poa annua Roots in Central European (Poland) and Antarctic Settings (King George Island)
Source: Microorganisms. 2021 Apr 12;9(4):811. doi: 10.3390/microorganisms9040811 (PMC8069831; doi:10.3390/microorganisms9040811)
Supplement: Supplementary file 1 [file microorganisms-09-00811-s001.zip › microorganisms-1161628-supplementary/Supplementary file 2.docx]

**Table S2.** Relative abundance heatmap of sequences identified on a family-rank taxonomic level. S – rhizospheric soil samples, R – root samples, P1-P2 – Central European (Poland) samples, P3-P5 – Antarctic samples (King George Island).

| **P1S** | **P1R** | **P2S** | **P2R** | **P3S** | **P3R** | **P4S** | **P4R** | **P5S** | **P5R** |  |
| --- | --- | --- | --- | --- | --- | --- | --- | --- | --- | --- |
| 1.99 | 0.52 | 0.71 | 0.17 | 2.35 | 0.36 | 0.92 | 0.08 | 3.90 | 0.62 | Acidimicrobiaceae |
| 4.48 | 1.05 | 1.60 | 0.30 | 0.72 | 0.38 | 0.41 | 0.05 | 1.76 | 0.68 | Blastocatellaceae |
| 4.23 | 1.12 | 1.49 | 0.68 | 1.11 | 1.27 | 0.42 | 0.40 | 0.66 | 1.28 | Bradyrhizobiaceae |
| 0.44 | 0.06 | 0.47 | 0.01 | 2.12 | 0.23 | 0.74 | 0.02 | 1.39 | 0.06 | Bryobacteraceae |
| 4.12 | 3.19 | 1.83 | 3.02 | 0.98 | 1.71 | 0.65 | 1.30 | 0.58 | 3.30 | Caulobacteraceae |
| 3.61 | 4.22 | 5.33 | 1.81 | 3.90 | 2.44 | 1.82 | 0.74 | 6.42 | 3.11 | Chitinophagaceae |
| 3.29 | 2.27 | 2.68 | 0.31 | 1.88 | 1.38 | 0.95 | 0.17 | 5.72 | 1.76 | Chthoniobacteraceae |
| 0.00 | 0.00 | 0.02 | 0.01 | 0.07 | 0.03 | 12.70 | 1.04 | 0.02 | 0.04 | Clostridiaceae |
| 3.27 | 7.25 | 2.55 | 7.29 | 2.35 | 4.55 | 2.03 | 1.24 | 3.21 | 4.20 | Comamonadaceae |
| 0.18 | 0.08 | 0.09 | 0.02 | 0.10 | 0.03 | 0.05 | 0.00 | 1.18 | 0.17 | Conexibacteraceae |
| 3.14 | 0.03 | 1.09 | 0.03 | 3.81 | 0.68 | 1.33 | 0.03 | 2.58 | 0.88 | CP011215_f |
| 3.02 | 2.73 | 0.93 | 2.82 | 0.82 | 1.19 | 0.75 | 0.91 | 0.46 | 1.60 | Cytophagaceae |
| 2.28 | 8.45 | 4.95 | 26.49 | 14.15 | 16.17 | 25.25 | 28.73 | 2.23 | 8.04 | Flavobacteriaceae |
| 2.21 | 1.34 | 0.80 | 0.15 | 0.73 | 0.31 | 0.41 | 0.04 | 0.77 | 0.13 | Gemmataceae |
| 0.58 | 0.14 | 1.92 | 0.12 | 1.15 | 0.46 | 0.61 | 0.03 | 3.41 | 0.54 | Gemmatimonadaceae |
| 0.71 | 0.07 | 1.16 | 0.13 | 1.07 | 0.13 | 0.77 | 0.06 | 1.11 | 0.52 | GQ396871_f |
| 0.15 | 0.23 | 1.81 | 0.44 | 0.28 | 0.12 | 0.24 | 0.19 | 0.41 | 0.27 | Hymenobacteraceae |
| 0.47 | 1.73 | 0.53 | 3.72 | 0.43 | 1.14 | 0.27 | 0.98 | 0.40 | 2.91 | Hyphomicrobiaceae |
| 2.12 | 0.22 | 0.58 | 0.19 | 0.41 | 0.18 | 0.19 | 0.05 | 0.52 | 0.23 | Iamiaceae |
| 0.39 | 0.20 | 0.62 | 0.28 | 0.63 | 0.08 | 0.71 | 0.06 | 1.18 | 0.27 | Intrasporangiaceae |
| 0.20 | 4.22 | 0.35 | 3.38 | 1.11 | 3.45 | 0.79 | 2.84 | 0.58 | 7.94 | Microbacteriaceae |
| 0.30 | 0.17 | 3.69 | 1.88 | 3.51 | 1.73 | 1.96 | 3.48 | 2.67 | 0.49 | Micrococcaceae |
| 1.77 | 0.76 | 0.37 | 1.06 | 0.04 | 0.22 | 0.01 | 0.01 | 0.02 | 0.07 | Micromonosporaceae |
| 0.55 | 0.23 | 0.53 | 0.03 | 1.01 | 0.16 | 0.33 | 0.01 | 0.40 | 0.17 | Micropepsaceae |
| 0.00 | 0.00 | 0.08 | 0.04 | 3.67 | 2.07 | 1.02 | 1.02 | 0.05 | 0.01 | Moraxellaceae |
| 1.69 | 2.11 | 1.95 | 1.78 | 0.29 | 0.08 | 0.31 | 0.06 | 1.55 | 0.75 | Nocardioidaceae |
| 0.62 | 0.17 | 0.40 | 0.07 | 2.07 | 0.68 | 0.70 | 0.04 | 0.96 | 0.26 | Opitutaceae |
| 0.48 | 1.51 | 4.51 | 4.97 | 2.64 | 2.31 | 8.30 | 20.98 | 1.51 | 4.14 | Oxalobacteraceae |
| 0.92 | 0.67 | 1.30 | 0.12 | 2.35 | 0.61 | 0.83 | 0.05 | 3.76 | 0.58 | PAC000016_f |
| 0.01 | 0.04 | 0.02 | 0.02 | 0.32 | 1.27 | 0.14 | 0.01 | 0.23 | 0.29 | PAC000380_f |
| 0.43 | 0.01 | 0.41 | 0.00 | 0.75 | 0.04 | 0.29 | 0.01 | 0.98 | 0.07 | PAC000536_f |
| 0.19 | 0.00 | 0.73 | 0.01 | 0.69 | 0.04 | 0.38 | 0.00 | 1.17 | 0.18 | PAC000624_f |
| 0.39 | 0.04 | 0.56 | 0.03 | 1.38 | 0.15 | 0.20 | 0.01 | 1.88 | 0.17 | PAC001907_f |
| 1.63 | 0.01 | 1.14 | 0.01 | 1.65 | 0.17 | 1.16 | 0.03 | 1.81 | 0.07 | Pedosphaera_f |
| 6.15 | 0.91 | 2.91 | 0.28 | 1.60 | 0.31 | 0.84 | 0.09 | 0.93 | 0.17 | Planctomycetaceae |
| 0.79 | 1.90 | 0.39 | 0.70 | 0.54 | 1.29 | 0.23 | 0.26 | 1.23 | 1.53 | Polyangiaceae |
| 0.62 | 1.85 | 1.96 | 6.72 | 1.13 | 25.82 | 7.42 | 22.79 | 0.12 | 1.38 | Pseudomonadaceae |
| 1.61 | 0.20 | 0.18 | 0.05 | 0.00 | 0.00 | 0.01 | 0.00 | 0.04 | 0.04 | Pseudonocardiaceae |
| 0.77 | 0.02 | 0.44 | 0.01 | 0.40 | 0.01 | 0.10 | 0.00 | 1.09 | 0.03 | Pyrinomonadaceae |
| 0.37 | 6.92 | 0.28 | 6.24 | 0.08 | 0.82 | 0.08 | 1.32 | 0.05 | 4.93 | Rhizobiaceae |
| 0.30 | 0.97 | 0.21 | 0.15 | 0.38 | 0.56 | 0.22 | 0.16 | 0.17 | 0.35 | Rhodobacteraceae |
| 0.51 | 0.90 | 0.19 | 0.25 | 0.40 | 1.79 | 0.31 | 0.26 | 0.41 | 2.45 | Roseiflexaceae |
| 0.31 | 0.64 | 1.67 | 0.87 | 1.31 | 1.69 | 4.30 | 0.93 | 0.98 | 1.53 | Saccharimonas_f |
| 0.43 | 0.04 | 0.60 | 0.04 | 1.88 | 0.68 | 0.61 | 0.03 | 0.61 | 0.20 | Saprospiraceae |
| 1.30 | 0.71 | 0.86 | 0.04 | 0.72 | 0.21 | 0.28 | 0.01 | 0.16 | 0.04 | Sinobacteraceae |
| 0.31 | 0.03 | 0.47 | 0.01 | 1.53 | 0.24 | 0.54 | 0.01 | 0.43 | 0.02 | Solibacteraceae |
| 1.57 | 8.11 | 2.13 | 6.84 | 0.80 | 4.11 | 2.21 | 3.13 | 1.15 | 10.24 | Sphingobacteriaceae |
| 1.54 | 9.26 | 3.72 | 3.32 | 2.02 | 3.21 | 2.04 | 2.36 | 5.26 | 15.29 | Sphingomonadaceae |
| 1.27 | 0.44 | 2.69 | 0.14 | 1.48 | 0.22 | 0.94 | 0.04 | 1.51 | 0.20 | Tepidisphaeraceae |
| 1.22 | 0.13 | 0.48 | 0.05 | 0.04 | 0.03 | 0.04 | 0.00 | 0.15 | 0.08 | Thermoleophilaceae |
| 1.84 | 1.33 | 1.56 | 1.15 | 1.47 | 0.64 | 1.36 | 0.28 | 1.36 | 0.96 | Verrucomicrobiaceae |
| 1.19 | 0.89 | 4.63 | 0.24 | 2.43 | 0.37 | 0.80 | 0.02 | 1.80 | 0.59 | Vicinamibacteraceae |
| 1.12 | 6.02 | 1.96 | 2.00 | 1.20 | 1.08 | 0.39 | 0.08 | 3.29 | 3.70 | Xanthomonadaceae |
| 0.02 | 0.02 | 0.00 | 0.00 | 0.00 | 1.86 | 0.08 | 1.44 | 0.00 | 0.00 | Yersiniaceae |
| P1S | P1R | P2S | P2R | P3S | P3R | P4S | P4R | P5S | P5R |  |
