# Supplementary material for: Bacterial Communities Associated with Poa annua Roots in Central European (Poland) and Antarctic Settings (King George Island)
Source: Microorganisms. 2021 Apr 12;9(4):811. doi: 10.3390/microorganisms9040811 (PMC8069831; doi:10.3390/microorganisms9040811)
Supplement: Supplementary file 1 [file microorganisms-09-00811-s001.zip › microorganisms-1161628-supplementary/Supplementary file 3.docx]

**Table S3.** Heatmap displaying *Poa annua* rhizosphere and root community responses on Biolog Ecoplates ; mean absorbance values from three replicates; color scale – absorbance values at 590nm. S – rhizospheric soil samples, R – root samples, P1-P2 – Central European (Poland) samples, P3-P5 – Antarctic samples (King George Island).

| **P1S** | **P1R** | **P2S** | **P2R** | **P3S** | **P3R** | **P4S** | **P4R** | **P5S** | **P5R** |  |
| --- | --- | --- | --- | --- | --- | --- | --- | --- | --- | --- |
| 0.553849 | 0.410491 | 0.605026 | 0.423824 | 0.279775 | 0.213346 | 0.314672 | 0.291975 | 0.290741 | 0.101743 | β-Methyl-D-Glucoside |
| 0.388251 | 0.415863 | 0.584289 | 0.303049 | 0.276394 | 0.220817 | 0.135727 | 0.235285 | 0.120823 | 0.135187 | D-Galactonic Acid γ-Lactone |
| 0.708439 | 0.54503 | 0.946189 | 0.898088 | 0.620724 | 0.786051 | 0.517246 | 0.67984 | 0.597551 | 0.128734 | L-Arginine |
| 0.307963 | 0.294077 | 0.370388 | 0.229282 | 0.258837 | 0.218988 | 0.172252 | 0.19242 | 0.155885 | 0.083423 | Pyruvic Acid Methyl Ester |
| 0.891593 | 0.444048 | 0.588114 | 0.572864 | 0.292432 | 0.337743 | 0.259678 | 0.629146 | 0.004588 | 0.000913 | D-Xylose |
| 0.50306 | 0.444606 | 0.522054 | 0.596134 | 0.352586 | 0.285253 | 0.162741 | 0.193238 | 0.253807 | 0.053299 | D-Galacturonic Acid |
| 0.840531 | 0.954092 | 0.657132 | 0.985522 | 0.642788 | 0.588191 | 0.433127 | 0.619359 | 0.24107 | 0.476017 | L-Asparagine |
| 0.61267 | 0.45215 | 0.657545 | 0.736574 | 0.531257 | 0.432802 | 0.357477 | 0.432064 | 0.259527 | 0.236763 | Tween 40 |
| 0.843864 | 0.443021 | 0.522791 | 0.01334 | 0.954709 | 1.003696 | 0.888198 | 1.001833 | 0.485041 | 1.003776 | i-Erythritol |
| 0.227198 | 0.002396 | 0 | 0 | 0.140332 | 0.235097 | 0.171467 | 0.095214 | 0.001481 | 0 | 2-Hydroxy Benzoic Acid |
| 0.641788 | 0.122351 | 0.768269 | 0.071777 | 0.33605 | 0.294105 | 0.497683 | 0.504733 | 0.173189 | 0.050021 | L-Phenylalanine |
| 0.755605 | 0.704263 | 0.559406 | 0.636077 | 0.436833 | 0.292257 | 0.085006 | 0.464431 | 0.236523 | 0.123133 | Tween 80 |
| 0.965277 | 1.001741 | 1.000439 | 1.000483 | 1.002242 | 0.89928 | 1.003063 | 0.998381 | 0.948683 | 0.830104 | D-Mannitol |
| 0.824871 | 0.112388 | 0.409096 | 0.141144 | 0.204543 | 0.143599 | 0.256178 | 0.297722 | 0.000267 | 0 | 4-Hydroxy Benzoic Acid |
| 0.562162 | 0.500067 | 0.87801 | 0.508863 | 0.474899 | 0.48572 | 0.23547 | 0.370356 | 0.387284 | 0.130041 | L-Serine |
| 0.754379 | 0.42782 | 0.763101 | 0.03924 | 0.884247 | 0.021226 | 0.804659 | 0.875356 | 0.978436 | 0.572884 | α-Cyclodextrin |
| 0.621522 | 0.639003 | 0.794044 | 0.704954 | 0.420996 | 0.379319 | 0.496384 | 0.398256 | 0.75428 | 0.277676 | N-Acetyl-D-Glucosamine |
| 0.619906 | 0.795856 | 0.68761 | 0.673298 | 0.399668 | 0.509105 | 0.255997 | 0.372206 | 0.210309 | 0.180622 | γ-Hydroxybutyric Acid |
| 0.252615 | 0.029092 | 0.139264 | 0.000384 | 0.521969 | 0.477412 | 0.310438 | 0.503683 | 0.201481 | 0 | L-Threonine |
| 0.641155 | 0.042448 | 0.657623 | 0.07086 | 0.425374 | 0.082782 | 0.63175 | 0.53395 | 0.141214 | 0.027905 | Glycogen |
| 0.446393 | 0.279256 | 0.441421 | 0.437299 | 0.330795 | 0.339494 | 0.278662 | 0.287705 | 0.176728 | 0.372448 | D-Glucosaminic Acid |
| 0.497728 | 0.271815 | 0.7 | 0.269709 | 0.29159 | 0.171401 | 0.090837 | 0.221263 | 0.170597 | 0 | Itaconic Acid |
| 0.467494 | 0.450305 | 0.498269 | 0.322601 | 0.32726 | 0.392529 | 0.387143 | 0.337384 | 0.255638 | 0.133216 | Glycyl-L-Glutamic Acid |
| 0.999961 | 0.742336 | 0.947106 | 0.660917 | 0.572017 | 0.441187 | 0.554839 | 0.607705 | 1.002181 | 0.434647 | D-Cellobiose |
| 0.314036 | 0.341711 | 0.362791 | 0.243163 | 0.171068 | 0.057393 | 0.133282 | 0.191957 | 0.123272 | 0.011743 | Glucose-1-Phosphate |
| 0.195144 | 0.000885 | 0.008475 | 0 | 0.001091 | 0 | 0.123629 | 0.151103 | 0.05284 | 0 | α-Ketobutyric Acid |
| 0.650156 | 0.050804 | 0.564199 | 0.26565 | 0.241601 | 0.051381 | 0.243102 | 0.335498 | 0.184033 | 0.195871 | Phenylethylamine |
| 0.943739 | 0.606704 | 0.659264 | 0.710817 | 0.725042 | 0.437062 | 0.889627 | 0.722687 | 0.646502 | 0.671618 | α-D-Lactose |
| 0.104379 | 0.083028 | 0.103514 | 0.110945 | 0.097058 | 0.088132 | 0.097709 | 0.189947 | 0.071008 | 0.021494 | D,L-α-Glycerol Phosphate |
| 0.279274 | 0.306057 | 0.751202 | 0.462701 | 0.204781 | 0.146693 | 0.107825 | 0.134075 | 0.153621 | 0.080622 | D-Malic Acid |
| 0.278126 | 0.402917 | 0.207416 | 0.341827 | 0.243677 | 0.291089 | 0.117606 | 0.231922 | 0.105741 | 0.15473 | Putrescine |
| P1S | P1R | P2S | P2R | P3S | P3R | P4S | P4R | P5S | P5R |  |
