# Supplementary material for: Bacterial Communities Associated with Poa annua Roots in Central European (Poland) and Antarctic Settings (King George Island)
Source: Microorganisms. 2021 Apr 12;9(4):811. doi: 10.3390/microorganisms9040811 (PMC8069831; doi:10.3390/microorganisms9040811)
Supplement: Supplementary file 1 [file microorganisms-09-00811-s001.zip › microorganisms-1161628-supplementary/Supplementary file 4.docx]

**Table S4.** Correlations of rhizospheric family-rank sequence abundance data and soil chem-istry. Only significant (*p* < 0.05) correlations are shown.

| **Row** | **Column** | **Cor** | ***p*** |
| --- | --- | --- | --- |
| Caulobacteraceae | Planctomycetaceae | 0.998453 | 7.3 × 10^−5^ |
| Gemmataceae | Iamiaceae | 0.997972 | 0.00011 |
| Micromonosporaceae | Pseudonocardiaceae | 0.995155 | 0.000404 |
| Planctomycetaceae | Verrucomicrobiaceae | 0.994822 | 0.000447 |
| Caulobacteraceae | Verrucomicrobiaceae | 0.990593 | 0.001094 |
| Bradyrhizobiaceae | Caulobacteraceae | 0.99041 | 0.001126 |
| Rhizobiaceae | Fe | −0.98971 | 0.001251 |
| Iamiaceae | Pseudonocardiaceae | 0.989624 | 0.001267 |
| Bradyrhizobiaceae | Planctomycetaceae | 0.989073 | 0.001369 |
| Cytophagaceae | Pseudonocardiaceae | 0.989041 | 0.001375 |
| Bradyrhizobiaceae | Micromonosporaceae | 0.988481 | 0.001481 |
| Cytophagaceae | Micromonosporaceae | 0.987919 | 0.001591 |
| Caulobacteraceae | Micromonosporaceae | 0.987654 | 0.001644 |
| Iamiaceae | Micromonosporaceae | 0.985793 | 0.002028 |
| Bradyrhizobiaceae | Verrucomicrobiaceae | 0.985477 | 0.002096 |
| Thermoleophilaceae | Salinity | −0.98499 | 0.002203 |
| Caulobacteraceae | Thermoleophilaceae | 0.984223 | 0.002373 |
| Rhizobiaceae | Salinity | −0.98352 | 0.002534 |
| Bradyrhizobiaceae | Iamiaceae | 0.983225 | 0.002602 |
| Bradyrhizobiaceae | Gemmataceae | 0.982958 | 0.002664 |
| Micromonosporaceae | Thermoleophilaceae | 0.982474 | 0.002778 |
| Planctomycetaceae | Thermoleophilaceae | 0.981946 | 0.002904 |
| Gemmataceae | Pseudonocardiaceae | 0.981621 | 0.002983 |
| Planctomycetaceae | Salinity | −0.98082 | 0.00318 |
| Blastocatellaceae | Iamiaceae | 0.979481 | 0.003517 |
| Micromonosporaceae | Planctomycetaceae | 0.97943 | 0.00353 |
| Bradyrhizobiaceae | Pseudonocardiaceae | 0.979144 | 0.003604 |
| Bradyrhizobiaceae | Cytophagaceae | 0.979044 | 0.00363 |
| Caulobacteraceae | Salinity | −0.97898 | 0.003646 |
| Gemmataceae | Micromonosporaceae | 0.976863 | 0.00421 |
| Salinity | Fe | 0.976613 | 0.004278 |
| Blastocatellaceae | Gemmataceae | 0.974592 | 0.004843 |
| Caulobacteraceae | Cytophagaceae | 0.972253 | 0.005525 |
| Bradyrhizobiaceae | Thermoleophilaceae | 0.97074 | 0.005982 |
| Caulobacteraceae | Pseudonocardiaceae | 0.96852 | 0.006673 |
| Cytophagaceae | Iamiaceae | 0.966907 | 0.007191 |
| Iamiaceae | Thermoleophilaceae | 0.966811 | 0.007222 |
| Pseudonocardiaceae | Thermoleophilaceae | 0.964739 | 0.007906 |
| Micromonosporaceae | Verrucomicrobiaceae | 0.962794 | 0.008567 |
| Verrucomicrobiaceae | Salinity | −0.96269 | 0.008603 |
| Caulobacteraceae | Iamiaceae | 0.962623 | 0.008626 |
| Cu | Fe | 0.96153 | 0.009005 |
| Rhizobiaceae | Cu | −0.96091 | 0.009222 |
| Cytophagaceae | Gemmataceae | 0.96037 | 0.009414 |
| Cytophagaceae | Planctomycetaceae | 0.960317 | 0.009433 |
| Blastocatellaceae | Thermoleophilaceae | 0.960007 | 0.009543 |
| Sinobacteraceae | Verrucomicrobiaceae | 0.958764 | 0.009989 |
| Thermoleophilaceae | Verrucomicrobiaceae | 0.958011 | 0.010263 |
| Iamiaceae | Planctomycetaceae | 0.9569 | 0.010671 |
| Planctomycetaceae | Pseudonocardiaceae | 0.956776 | 0.010717 |
| Caulobacteraceae | Gemmataceae | 0.955583 | 0.011162 |
| Gemmataceae | Thermoleophilaceae | 0.954513 | 0.011566 |
| Cytophagaceae | Verrucomicrobiaceae | 0.953021 | 0.012137 |
| Micromonosporaceae | Salinity | −0.95301 | 0.012142 |
| Blastocatellaceae | Pseudonocardiaceae | 0.952754 | 0.01224 |
| Gemmataceae | Planctomycetaceae | 0.951903 | 0.012571 |
| Blastocatellaceae | Micromonosporaceae | 0.950227 | 0.01323 |
| Bradyrhizobiaceae | Salinity | −0.94673 | 0.014642 |
| Planctomycetaceae | Rhizobiaceae | 0.944436 | 0.015591 |
| Cytophagaceae | Thermoleophilaceae | 0.943949 | 0.015795 |
| Iamiaceae | Verrucomicrobiaceae | 0.940052 | 0.01746 |
| Blastocatellaceae | Bradyrhizobiaceae | 0.940013 | 0.017477 |
| Gemmataceae | Verrucomicrobiaceae | 0.93995 | 0.017505 |
| Pseudonocardiaceae | Verrucomicrobiaceae | 0.938184 | 0.018278 |
| Rhizobiaceae | Thermoleophilaceae | 0.937933 | 0.018388 |
| Thermoleophilaceae | Fe | −0.93786 | 0.01842 |
| Caulobacteraceae | Rhizobiaceae | 0.937786 | 0.018453 |
| Sinobacteraceae | Cu | −0.93263 | 0.020779 |
| Planctomycetaceae | Fe | −0.93175 | 0.021184 |
| Rhizobiaceae | Verrucomicrobiaceae | 0.93115 | 0.021461 |
| Planctomycetaceae | Sinobacteraceae | 0.929328 | 0.022313 |
| Blastocatellaceae | Caulobacteraceae | 0.921882 | 0.0259 |
| Caulobacteraceae | Fe | −0.92045 | 0.026608 |
| Pseudonocardiaceae | Salinity | −0.91985 | 0.026911 |
| Blastocatellaceae | Planctomycetaceae | 0.918869 | 0.0274 |
| Salinity | Cu | 0.917599 | 0.028041 |
| Caulobacteraceae | Sinobacteraceae | 0.915808 | 0.028952 |
| Iamiaceae | Salinity | −0.91557 | 0.029072 |
| Verrucomicrobiaceae | Fe | −0.91433 | 0.029711 |
| Cytophagaceae | Salinity | −0.91331 | 0.030237 |
| Blastocatellaceae | Cytophagaceae | 0.902498 | 0.036008 |
| Bradyrhizobiaceae | Sinobacteraceae | 0.902101 | 0.036226 |
| Blastocatellaceae | Salinity | −0.9014 | 0.036614 |
| Gemmataceae | Salinity | −0.90091 | 0.036879 |
| Verrucomicrobiaceae | Cu | −0.89894 | 0.037973 |
| Rhizobiaceae | Sinobacteraceae | 0.897813 | 0.038606 |
| Sinobacteraceae | Salinity | −0.89024 | 0.042924 |
| Planctomycetaceae | Cu | −0.89017 | 0.042964 |
| Blastocatellaceae | Verrucomicrobiaceae | 0.88782 | 0.044336 |
| Micromonosporaceae | Rhizobiaceae | 0.887319 | 0.04463 |
| Bradyrhizobiaceae | Rhizobiaceae | 0.886228 | 0.045272 |

**Table S5.** Correlations between root endosphere family-rank sequence abundance data. Only significant (*p* < 0.05) correlations are shown.

| **Row** | **Column** | **Cor** | ***p*** |
| --- | --- | --- | --- |
| Blastocatellaceae | Vicinamibacteraceae | 0.996874 | 0.00021 |
| Chthoniobacteraceae | Micropepsaceae | 0.996119 | 0.00029 |
| Chitinophagaceae | Vicinamibacteraceae | 0.995009 | 0.000423 |
| Acidimicrobiaceae | Flavobacteriaceae | −0.9867 | 0.001838 |
| Blastocatellaceae | Chitinophagaceae | 0.985734 | 0.002041 |
| Chitinophagaceae | Polyangiaceae | 0.982827 | 0.002695 |
| Chthoniobacteraceae | Polyangiaceae | 0.98217 | 0.00285 |
| Micropepsaceae | Polyangiaceae | 0.979546 | 0.003501 |
| Chthoniobacteraceae | Flavobacteriaceae | −0.97596 | 0.004459 |
| Polyangiaceae | Vicinamibacteraceae | 0.967201 | 0.007095 |
| Caulobacteraceae | Pseudomonadaceae | −0.96561 | 0.007615 |
| Flavobacteriaceae | Micropepsaceae | −0.96534 | 0.007705 |
| Flavobacteriaceae | Polyangiaceae | −0.96181 | 0.008909 |
| Chitinophagaceae | Chthoniobacteraceae | 0.956697 | 0.010746 |
| Chthoniobacteraceae | Vicinamibacteraceae | 0.952665 | 0.012274 |
| Caulobacteraceae | Sphingobacteriaceae | 0.946485 | 0.014741 |
| Blastocatellaceae | Polyangiaceae | 0.944202 | 0.015689 |
| Chitinophagaceae | Micropepsaceae | 0.943312 | 0.016064 |
| Pseudomonadaceae | Rhizobiaceae | −0.93971 | 0.017609 |
| Caulobacteraceae | Intrasporangiaceae | 0.935556 | 0.019447 |
| Pseudomonadaceae | Sphingobacteriaceae | −0.93342 | 0.020418 |
| Micropepsaceae | Vicinamibacteraceae | 0.932666 | 0.020761 |
| Acidimicrobiaceae | Chthoniobacteraceae | 0.930267 | 0.021872 |
| Iamiaceae | Oxalobacteraceae | −0.92997 | 0.022011 |
| Blastocatellaceae | Chthoniobacteraceae | 0.929952 | 0.02202 |
| Acidimicrobiaceae | Polyangiaceae | 0.928574 | 0.022668 |
| Caulobacteraceae | Rhizobiaceae | 0.922453 | 0.025619 |
| Chitinophagaceae | Flavobacteriaceae | −0.92216 | 0.025764 |
| Flavobacteriaceae | Vicinamibacteraceae | −0.91616 | 0.028774 |
| Acidimicrobiaceae | Micropepsaceae | 0.912786 | 0.03051 |
| Blastocatellaceae | Micropepsaceae | 0.904541 | 0.034893 |
| Intrasporangiaceae | Pseudomonadaceae | −0.89821 | 0.038381 |
| Bradyrhizobiaceae | Flavobacteriaceae | −0.89149 | 0.042204 |
| Blastocatellaceae | Flavobacteriaceae | −0.89019 | 0.042952 |
| Acidimicrobiaceae | Chitinophagaceae | 0.888743 | 0.043797 |
| Acidimicrobiaceae | Bradyrhizobiaceae | 0.886093 | 0.045352 |
| Acidimicrobiaceae | Vicinamibacteraceae | 0.881299 | 0.048209 |
| Bradyrhizobiaceae | Polyangiaceae | 0.880472 | 0.048708 |
